# Metal–Organic Enzyme Nanogels as Nanointegrated Self-Reporting Chemobiosensors

**DOI:** 10.1021/acsami.2c04385

**Published:** 2022-06-08

**Authors:** Daniel Sánchez-deAlcázar, Andoni Rodriguez-Abetxuko, Ana Beloqui

**Affiliations:** † POLYMAT and Department of Applied Chemistry, Faculty of Chemistry, University of the Basque Country UPV/EHU, E-20018 Donostia-San Sebastián, Spain; ‡ CIC nanoGUNE, Basque Research and Technology Alliance (BRTA), Tolosa Hiribidea 76, E-20018 Donostia-San Sebastián, Spain; § IKERBASQUE, Basque Foundation for Science, Plaza Euskadi 5, E-48009 Bilbao, Spain

**Keywords:** chemobiosensor, polymeric scaffolds, supramolecular assembly, fluorometric biosensor, smart hybrid nanomaterials, lanthanides

## Abstract

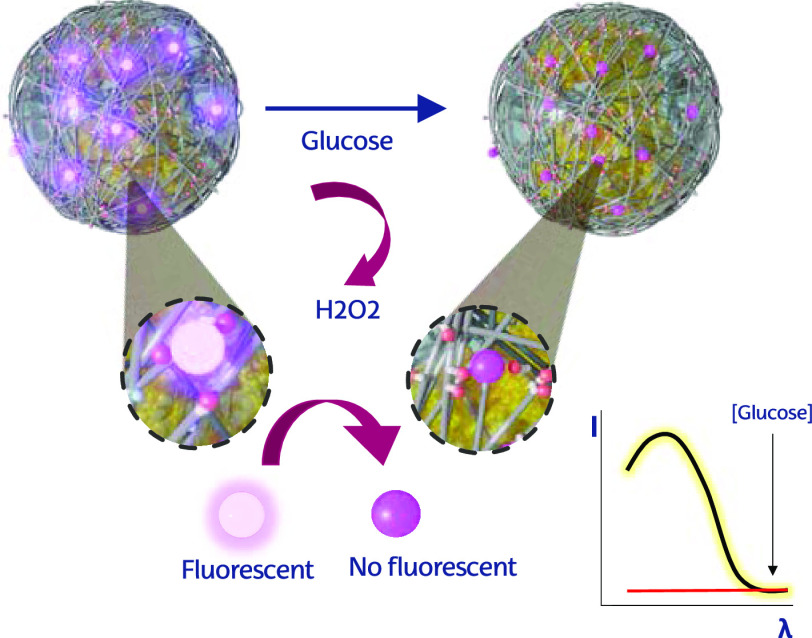

A fluorometric glucose biosensor based on fine-tuned chemoenzymatic nanohybrids is herein proposed. The successful integration of an engineered glucose oxidase enzyme and an optically responsive polymeric nanogel in a single entity has led to the fabrication of a highly efficient glucose chemobiosensor. The optical responsiveness has been achieved by the loading of preactivated polymeric hydrogel with fluorescent lanthanide, i.e., cerium (III), cations. A comprehensive investigation of the responsiveness of the biomaterial revealed the interplay between the oxidation state of the cerium lanthanide and the fluorescence emission of the polymer. Finally, a full structural, chemical, and biochemical characterization of the reported system supports the chemobiosensors as robust, specific, and sensitive materials that could be utilized to faithfully quantify the amount of glucose in tear fluids.

## Introduction

Polymers have been applied as synthetic scaffolds for the embedment and the shielding of labile functional entities such as biomacromolecules, organic catalysts, or inorganic nanoparticles.^1−4^ The configuration of these hybrid (bio)materials stimulates the intrinsic features of the bio-entities such as the operational stability or the reusability. Indeed, there are several reported examples in which the improved attributes gained throughout the combination of enzymes and polymers are harnessed in nonconventional biocatalytic processes (e.g., reactions that proceed in the presence of organic solvents or at high temperatures) or in technological applications.^5−7^ Alternatively, in this work, we are interested in the utilization of polymeric supramolecular conformations as 3D architectures to confine two distinct functional entities, i.e., enzymes and inorganic reporters, which are eventually contributing to the same concurrent process, namely, the detection and quantification of glucose.

Following classical methodologies to detect and quantify analytes with high sensitivity and selectivity, physicochemical reporters, i.e., fluorescent probes or materials, are separately co-added with the enzyme, which will transform the analyte of interest in the test sample.^8−10^ Conversely, we envisioned the co-localization of both the active materials into the nanospace through the design of convenient polymeric scaffolds as a potential approach for the fabrication of promising chemoenzymatic biosensors. Specifically, we propose core–shell polymer-based nanomaterials. The enzyme, which is localized in the core, recognizes and transforms the analyte into a reaction intermediate that is released to the environment throughout the polymeric shell in which the reporter is accommodated. This configuration definitely seeks a controlled and close positioning of the functional entities for the sake of an efficient multistep procedure that requires the minimization of mass-transport issues. To demonstrate the potential use of this configuration, we have designed a fluorescence-based chemobiosensor for the detection of glucose at low concentrations, in the ppm range.

Fluorescence emitting molecules have been chosen as reporters due to their exceptional compatibility and sensitivity.^11^ Specifically, lanthanides have received interest in the fabrication of fluorescent sensors as a result of their excellent photochemical stability, large stokes shift, and long fluorescent lifetime.^12−15^ Among them, Ce-based nanomaterials have been exploited as sensors for the detection of organic molecules, metal ions, inorganic salts, and biological molecules.^8,10,16−18^ Thus, we propose the fabrication of a supramolecular system in which distinct components, i.e., glucose oxidase (GOx) enzyme and Ce (III), are integrated in a unique polymeric entity and work together in a concurrent manner for the detection and quantification of glucose throughout optical readout.

## Materials and Methods

### Synthesis of Single Enzyme Nanogels (SEN)

Phosphate-decorated GOx nanogels (pGOx) were synthesized following a protocol based on the conditions described before.^19^ GOx enzyme (20 μM) was mixed with acrylamide (AA/GOx 600:1, n/n), *N*,*N*′-methylenebisacrylamide (MBAAm, MBAAm/GOx 400:1, n/n), monoacryloxyethyl phosphate (MAEP, MAEP/GOx 0:1, 100:1, 200:1, 400:1, n/n), and ammonium persulfate (APS/protein 400:1, n/n). Sucrose (5%, w/v) and DMSO (10%, v/v) were also added to the reaction. This mixture was deoxygenated by bubbling N_2_ for 45 min. Polymerization reaction started upon the addition of tetramethylethylenediamine (TEMED, APS/TEMED 2:1, w/w). The reaction was kept under N_2_ and shaken at room temperature for 2 h. GOx nanogels were dialyzed (MWCO of 10 kDa) against Tris-HCl buffer (5 mM pH 7.0) to remove low-molar-mass reagents.

### Synthesis of Ln-laden Nanogels

Lanthanide-laden pGOx nanogels (Ln@pGOx) were synthesized by mixing pGOx nanogels (500 μL, 1.25 μM) prepared in Tris-HCl buffer (5 mM, pH 8.0) with lanthanide nitrate salts, i.e., Ce(NO_3_)_3_·6H_2_O, Tb(NO_3_)_3_·5H_2_O, or Pr(NO_3_)_3_·6H_2_O (final concentration of 0.2 mM). The mixture was stirred for 2 h at room temperature. Thereafter, the excess of cerium was removed using a Sephadex PD10 column. An additional step was required to test the use of antenna ligands. In this particular case, 1 mM bypyridine or ATP was added to the final solution of Ce@pGOx and incubated for 2 h at room temperature. Then, the excess ligand was removed by dialysis (MWCO of 10 kDa) and by using a Sephadex PD10 column.

### Instrumentation—Sodium Dodecyl Sulfate–Polyacrylamide Gel Electrophoresis (SDS-PAGE)

SDS-PAGE was performed using 10% acrylamide gels on Bio-Rad Mini-PROTEAN tetra system. pGOx or free GOx (5 μg) were mixed with 5 μL of loading buffer (4×) and heated at 92 °C for 5 min. The sample was loaded onto electrophoresis gel and run at a constant voltage of 100 mV for 1 h. Afterward, the gel was stained with Imperial protein stain (Thermo Scientific) for 1 h and destained overnight with ultrapure water.

### Instrumentation—Size Exclusion Chromatography (SEC)

Gel filtration chromatography was performed using AKTA GO Fast Protein Liquid Chromatography (FPLC) equipment (Cytiva). The samples were injected into a Superdex 200 Increase 10/300 GL size exclusion chromatography column (Cytiva) and were run at 0.5 mL min^–1^ in PBS. A loop of 100 μL was used, and 50 μL was injected in each run.

### Instrumentation—Attenuated Total Reflectance–Fourier Transform Infrared (ATR-FTIR) Spectroscopy

Briefly, 8 μL (GOx concentration from 43.7 to 62.5 μM) was deposited and dried on a silicon wafer. The FTIR spectra were measured with a PerkinElmer Frontier spectrometer equipped with an ATR sampling stage. All spectra were measured with 20 scans in the 600–4000 cm^–1^ wavenumber window with a 4 cm^–1^ resolution. The baseline was removed for spectra representation. Each sample was measured three times, and the results were averaged.

### Instrumentation—Dynamic Light Scattering (DLS) and ζ-Potential Measurements

DLS and ζ-potential measurements were performed on a Malvern Zetasizer Nano ZS. Proteins and polymer hybrids (3.1 μM) were prepared in phosphate buffer (30 mM, pH 6.0) and filtered through 0.22 μm cutoff membranes. Experiments were performed at 22 °C, and 13 readouts were taken in three independent measurements for each sample. ζ-Potential measurements were performed in phosphate buffer (30 mM, pH 8.0) containing KCl salts (10 mM), using a final nanogel concentration of 6.25 μM.

### Instrumentation—Circular Dichroism (CD)

Circular dichroism (CD) spectra were recorded with a Jasco J-815CD spectrometer. CD spectra were acquired in a 1 mm pathlength quartz cuvette. All CD spectra were recorded with a bandwidth of 1 nm at 1 nm increments and 10 s average time over a wavelength range of 190–260 nm. The sample was prepared in Tris-HCl buffer (5 mM, pH 7.0) at 1.25 μM.

The thermal denaturation experiment was performed in a 0.1 cm pathlength quartz cuvette in Tris-HCl buffer (5 mM, pH 7.0). The denaturation curves were monitored by following the CD signal at a wavelength of 222 nm as a function of temperature from 20 to 90 °C.

### Instrumentation—Inductively Coupled Plasma Mass Spectrometry (ICP-MS)

Ce@pGOx (10, 100, and 500 μL) was mixed with 990, 900, and 500 μL of 3% HNO_3_, respectively, and incubated at 90 °C in an oil bath for 1h. The Ce concentration was determined using iCAP-Q ICP-MS (Thermo Scientific, Bremen, Germany) equipped with an auto-sampler ASX-500 (CETAC Technologies, Omaha). Data were monitored using the software Qtegra v2.6 (Thermo Fisher, Bremen, Germany) utilizing the ^140^Ce and ^193^Ir isotopes as internal standards.

### Instrumentation—Fluorometer

The fluorescence spectra were recorded with an FP6600 spectrofluorometer (Jasco). A black quartz cuvette of 10 mm pathlength was used. The experiments were performed at an excitation wavelength of 310 nm using a bandwidth of 10 mm and data pitch of 2 nm.

For glucose-sensing experiments, nanogels (200 μL, 0.6 μM) were placed in the cuvette and a range of concentrations, i.e., 0, 0.5, 1.75, 5, 10, 15, 20, 45, 70, and 95 μM, of glucose were added, with the stabilization time among measurements set to 30 min. The emission spectra were recorded for each concentration over a wavelength window of 330–500 nm. The limit of detection (LOD) and limit of quantification (LOQ) were calculated as follows
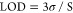
1
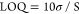
2where “*S*” is the slope of the calibration curve and ‘σ’ is the standard deviation of 10 blank samples, which is calculated using the following formula
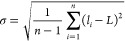
3where “li” is the fluorescence intensity of each blank sample (*I* = 1–10) and “*L*” is the average fluorescence intensity of 10 blank samples (*n* = 10).

### Instrumentation—X-ray Photoelectron Spectroscopy (XPS)

XPS experiments were performed in a SPECS Sage HR 100 spectrometer with a nonmonochromatic X-ray source (magnesium Kα line of 1253.6 eV energy and 252 W), placed perpendicular to the analyzer axis, and calibrated using the 3d_5/2_ line of Ag with a full width at half-maximum (FWHM) of 1.1 eV. The selected resolution for the spectra was 15 eV of pass energy and 0.15 eV/step. All measurements were made in an ultrahigh vacuum (UHV) chamber at a pressure of around 8 × 10^–8^ mbar. An electron flood gun was used to neutralize for charging.

### Instrumentation— Scanning Electron Microscopy (SEM)

Morphological characterization was done with a scanning electron microscope (FEI, Helios NanoLab 450S) at 5.0 kV and a current of 0.2 nA. The working distance was 3 mm. The samples were prepared using the spin-coating technique. Briefly, one drop of 20 μL (0.6 μM in water) was deposited in a silicon wafer and spin-coated at 30 rps for 1 min.

### Catalytic Activity Measurements

Standard activity assays were performed in 96-well plates in a Biotek Synergy Neo2 plate reader.

Activity measurements of plain GOx and pGOx were carried out using 3.2 μg of enzyme, 5.2 μg of HRP, 80 mM glucose in 30 mM sodium phosphate (150 μL, 30 mM, pH 6.0), and 1 mM 2,2′-azino-bis(3-ethylbenzothiazoline-6-sulfonic acid) (ABTS) at 42 °C.

Activity measurements were performed using 10 μg of Ce@pGOx, 50 mM glucose in 50 mM sodium phosphate (150 μL, 50 mM, pH 6.0), and 1 mM 2,2′-azino-bis(3-ethylbenzothiazoline-6-sulfonic acid) (ABTS) at 37 °C. The color development from oxidized ABTS was monitored at 416 nm. A molar extinction coefficient of 36,000 M^–1^ cm^–1^ was used for the calculations.

### Preparation of Biological Samples

BSS Distra-sol balanced salt solution (Ophcon) was used as artificial human being tears with the following composition: NaCl 1.3 gL^–1^, CH_3_COONa·3H_2_O 0.78 gL^–1^, Na_3_C_6_H_5_O_7_·2H_2_O 0.34 gL^–1^, KCl 0.15 gL^–1^, CaCl_2_·2H_2_O 0.096 gL^–1^, MgCl_2_·6H_2_O 0.06 gL^–1^.

## Results and Discussion

**Scheme 1:**
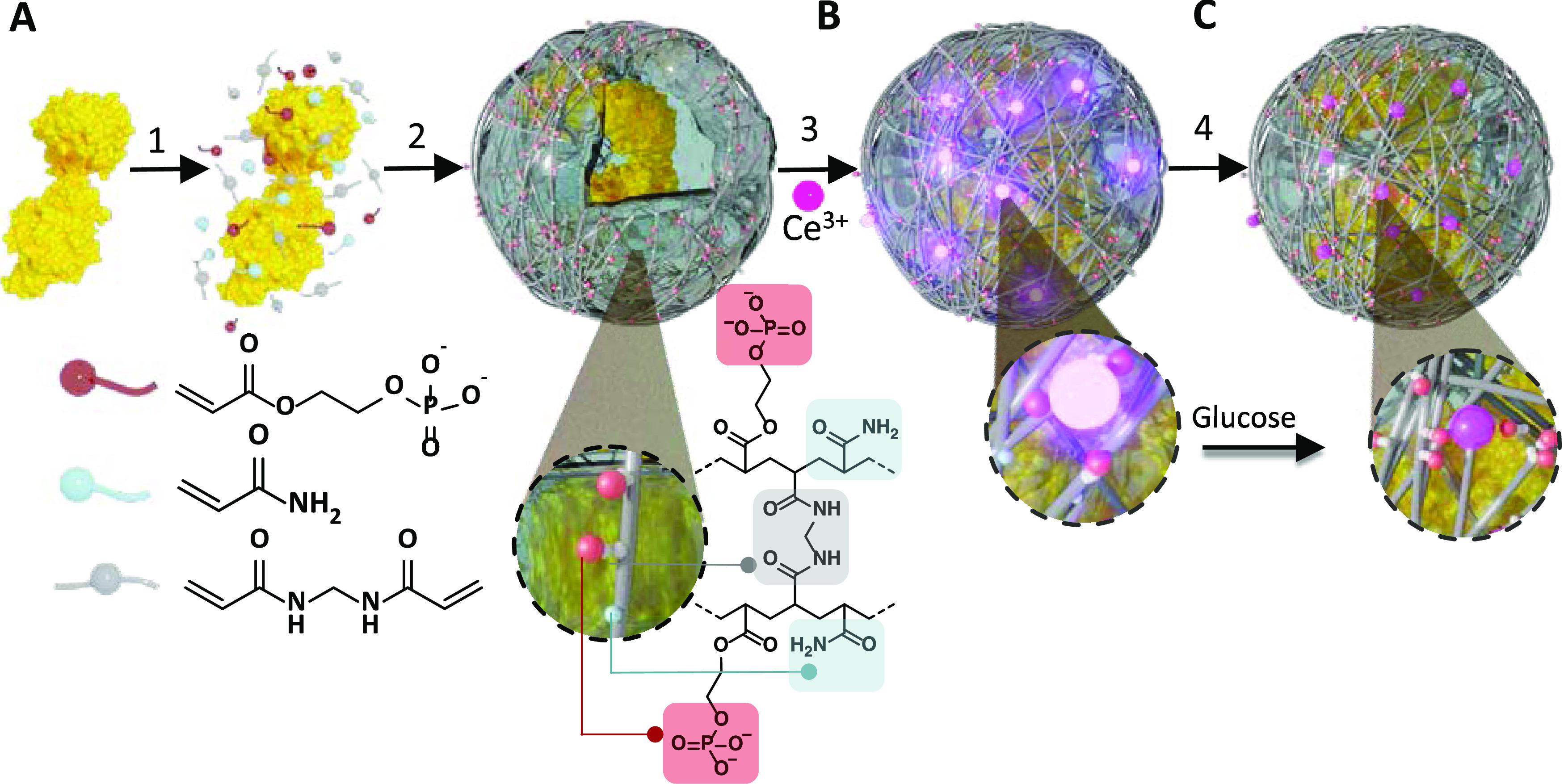
Proposed Workflow for the Synthesis of the Chemobiosensor and the Detection of Glucose (A) First (steps 1 and 2), GOx enzyme is encapsulated within a thin polyacrylamide network decorated with ethyl phosphate groups throughout a radical polymerization reaction that takes place on the surface of the protein (step 2). (B) Thereafter, enzyme nanogels (pGOx) are loaded with Ce (III) cations (step 3), which remain coordinated to the phosphate groups of the polymeric component, giving rise to a fluorescent pGOx (named Ce@pGOx). (C) Finally, in the presence of glucose (step 4), GOx enzyme will produce an equimolar amount of hydrogen peroxide, which quenches the fluorescence of the system.

We aim to fabricate a self-reporting glucose chemobiosensor within an integrated hybrid nanosystem. As illustrated in Scheme 1A, our approach entails the fabrication of reactive glucose oxidase nanogels (referred to as pGOx) throughout the deposition of a very thin but functional polymeric layer, with hanging phosphate groups on the surface of the enzyme. The fine tuning of these nanogels allows their combination with inorganic, *i.e.*, metal cations, or metal–organic, *i.e.*, iron porphyrins, compounds for their potential use as heterogeneous catalysts or chemoenzymatic nanoreactors.^20^ Herein, we envisioned the decoration of the polymeric mesh with fluorescent lanthanides, in particular, with cerium cations,^21,22^ throughout the interaction with the phosphate groups distributed along the shell of the hybrid (Scheme 1B).

### Synthesis and Characterization of the Functional Enzyme Nanogels

The synthesis of pGOx was performed following a protocol adapted from previous works (see the Materials and Methods section for further details). We hypothesized that achieving a higher content of phosphate groups within the polymeric network would result in nanomaterials with better photoluminescent features due to higher Ce loads. Therefore, we sought for the maximum incorporation of monoacryloxyethyl phosphate (MAEPm) monomer in the shell, while preserving the stability and solubility of the nanogels. A range of increased MAEPm/GOx molar ratios (*n*/*n*, from 0 to 400) were tested, giving rise to the samples listed in Table 1 (pGOx_0 to pGOX_3). The successful formation of the nanogels was confirmed by dynamic light scattering (DLS), protein electrophoresis, and fast protein liquid chromatography (FPLC) (Figures S1 and S2). DLS measurements evidenced the formation of nanogels with a hydrodynamic diameter from 9 to 13 nm. The largest nanogel samples were achieved at the highest MAEPm concentrations (12.87 nm for pGOx_3 sample). Considering the experimental hydrodynamic ratio of 8.55 ± 0.21 nm measured for unmodified GOx, a polymeric shell of ca. 0.42, 0.24, 0.88, and 2.16 nm thicknesses was calculated for samples pGOx_0, pGOx_1, pGOx_2, and pGOx_3, respectively. A molar ratio that exceeded 400 was also tested, i.e., MAEPm/GOx of 800, yet an uncontrolled polymerization that led to the formation of protein–polymer aggregates was achieved.

**Table 1:** Samples Synthesized in This Work and Their Respective Values Obtained for the Hydrodynamic Diameter Size and ζ-Potential

sample	monomer/protein ratio (n/n)	size (nm)	ζ-potential (mV)
pGOX_0	0	9.40 ± 0.45	–11.97 ± 1.32
pGOX_1	100	9.03 ± 1.18	–13.43 ± 2.54
pGOX_2	200	10.32 ± 1.21	–18.12 ± 2.72
pGOX_3	400	12.87 ± 0.94	–21.78 ± 1.97

**Figure 1:**
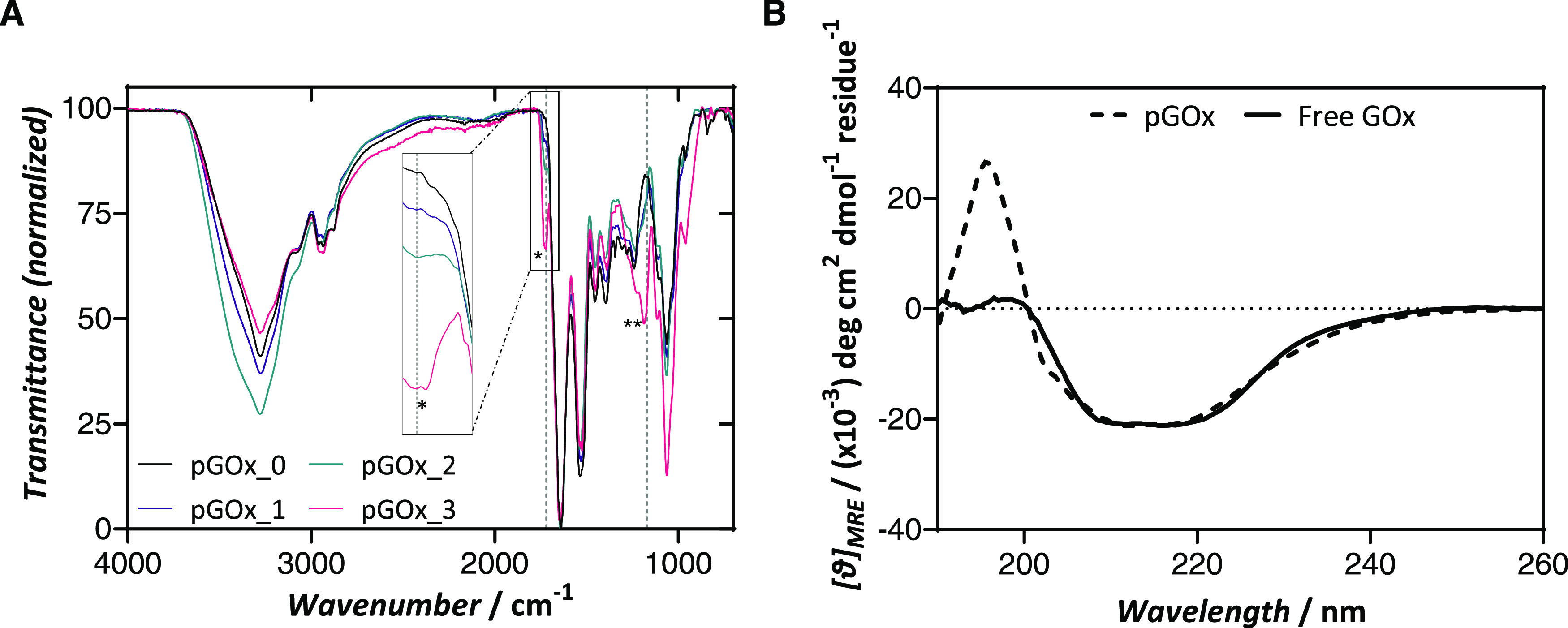
Characterization of pGOx nanogels. (A) ATR-FTIR spectra of nanogel samples. Characteristic bands, 1723 cm^–1^ (* and inset zoom in) and 1170 cm^–1^ (**), reveal the presence of phosphates within the pGOx_1 to 3 nanogels. (B) Circular dichroism spectra in the far-UV spectrum of unmodified and encapsulated GOx enzyme.

Both the chemical composition and the morphological features of the nanogels were ascertained using spectroscopic and microscopy tools, respectively. The insertion of ethyl phosphate groups within the polymeric network was evidenced by ζ-potential measurements and by attenuated total reflection–Fourier transform infrared (ATR-FTIR) spectroscopy. ζ-Potential measurements disclosed a correlation between the charge of the surface and the content of phosphate groups in the polymeric shell. While the nanogels that lacked MAEPm showed ζ-potential values of −11.97 mV, samples prepared with MAEPm showcased a progressive decrease in absolute ζ-potential values, reaching the maximum, i.e., −21.78 mV, when a molar excess of 400 was added to the synthesis procedure (Table 1). The insertion of phosphate groups is also evidenced in the FTIR spectra (Figure 1A). A band corresponding to the symmetric stretching vibration of the carbonyl group of the acrylate ester, i.e., 1723 cm^–1^, can be identified only in MAEPm containing samples (pGOx_1 to pGOx_3).^23,24^ Same samples show a differential shoulder that arises at 1150 cm^–1^, which is typically attributed to the antisymmetric stretching of phosphate groups.^25,26^

Remarkably, circular dichroism measurements carried out in the far-UV confirmed that the secondary structure of the protein was not affected upon polymerization reaction (Figure 1B). Moreover, thermal denaturation experiments monitored by circular dichroism indicated that the protein preserved the secondary structure and, in turn, its stability, along the temperature ramp, from 20 to 90 °C (Figure S3). In contrast, unprotected enzyme completely lost the dichroic signal at ca. 80 °C, reaching the melting temperature (*T*_m_) below 60 °C. Such improvement in thermal stability can be explained by the sheltering effect of the polymer that wraps the protein.^6,7,27^ Also, the catalytic activity of the pGOx nanogels was assessed and compared with unmodified GOx. As expected,^27−29^ the specific activity was not affected upon nanogel formation (Figure S4).

### Synthesis and Characterization of the Fluorescent Chemobiosensors

**Figure 2:**
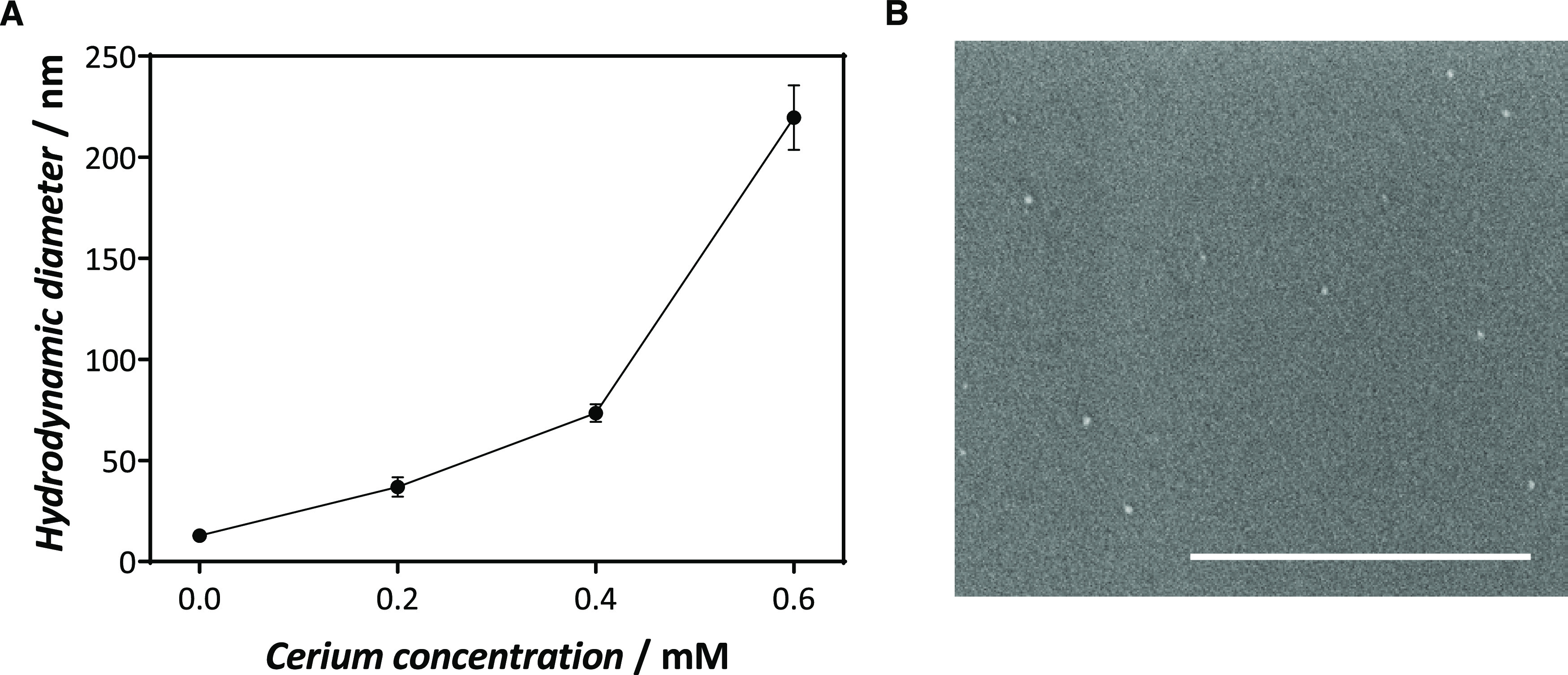
Characterization of Ce@pGOx_3 hybrids. (A) Hydrodynamic diameters measured by DLS for the hybrids assayed. (B) SEM pictogram of Ce@pGOx_3 hybrid synthesized from 0.2 mM of Ce^3+^ (scale bar: 400 nm).

Next, integrated chemobiosensors were assembled using pGOx nanogels and cerium nitrate salt. A range of cerium concentrations, from 0.1 to 0.8 mM, were mixed with pGOx_3 sample for 2 h at room temperature, resulting in Ce@pGOx hybrids (see the Materials and Methods section for further details). Inductively coupled plasma mass spectrometry (ICP-MS) measurements showcased a Ce(III) content from 237.5 ± 10.4 to 263.7 ± 11.2 Ce (III) ions per nanogel (Table S1), which means an estimated retention of ca. 76% of the seeded Ce (III). Further, we monitored the size of the hybrids by DLS at increasing concentrations of Ce (III) (Figure 2A). The size and the morphology of the particles were confirmed by scanning electron microscopy (SEM) (Figures 2B and S5). SEM images of Ce@pGOx_3 hybrids synthesized with 0.2 mM of Ce^3+^ showed monodisperse particles (9.94 ± 1.74 nm). However, we observed by DLS a higher hydrodynamic diameter (36.94 ± 4.8 nm) for the same sample. This might be explained by the spontaneous assembly of small clusters of a few nanogels in solution. Indeed, the addition of high concentrations of cerium (>0.4 mM) led to the complete aggregation of Ce@pGOx hybrids, which eventually precipitated as aggregates of ca. 200 nm (Figure S6). The excess of cerium might “bridge” individual nanogels, triggering the internanogel crosslinking due to the high affinity of phosphate groups toward cerium cations. In a previous work, we demonstrated a similar effect in the synthesis of metal–organic enzyme aggregates (MOEAs).^30^

**Figure 3:**
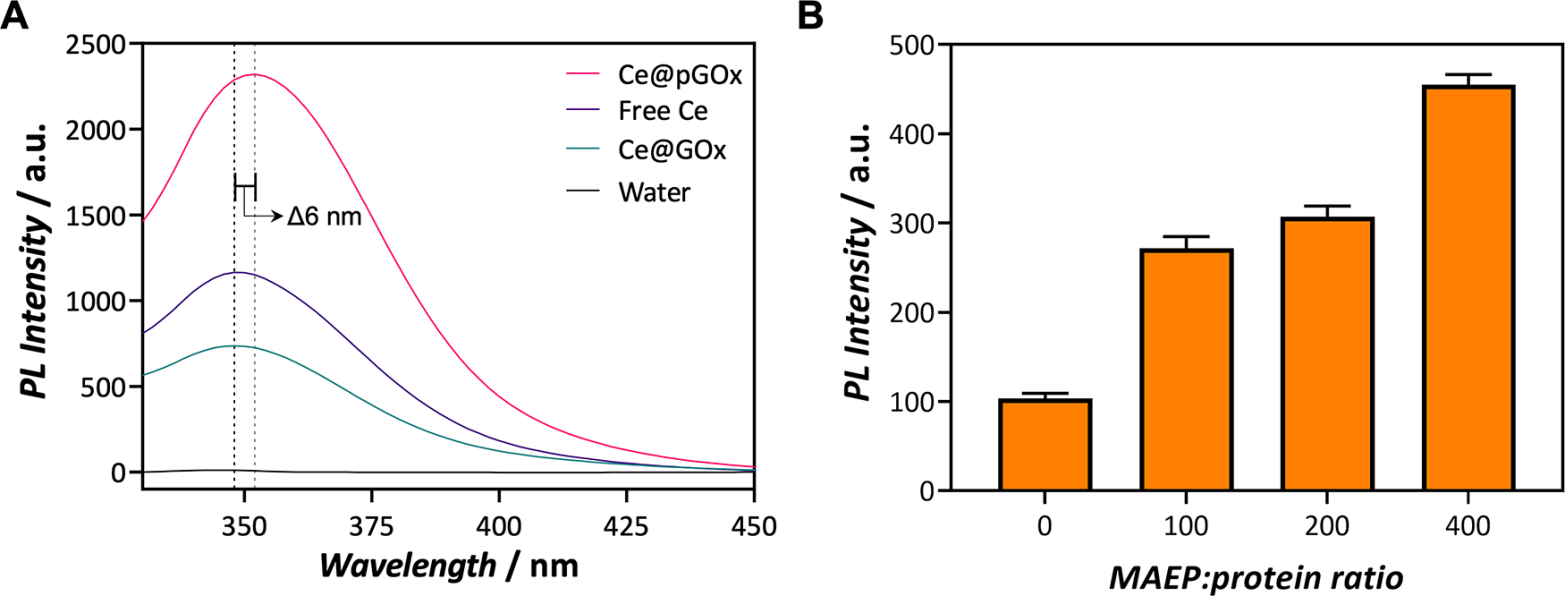
Photoluminescence analysis of the hybrids (λ_exc_ 310 nm). (A) Fluorescence emission spectra of the hybrid nanogels synthesized with and without phosphate groups (Ce@pGOx and Ce@GOx, respectively) and a free cerium solution at 0.2 mM. (B) Fluorescence intensity values recorded for Ce@GOx_0-3 samples assembled with 0.2 mM of Ce(III).

Next, the fluorescence of the hybrids was assessed. Upon excitation at 310 nm, Ce@pGOx nanogels displayed a maximum emission peak centered at 354 nm. Compared with cerium nitrate aqueous solution, a redshift of *ca.* 6 nm in the emission peak was observed (peak maxima at 348 vs 354 nm, for free Ce(III) and phosphate-coordinated Ce(III) samples, respectively), which again confirms the phosphate–Ce interaction.^31^ The synthesis conditions were demonstrated to be highly reproducible, with a standard deviation of the intrinsic fluorescence of 2% among synthesized batches (Figure S7). Remarkably, in comparison with the control sample synthesized without MAEPm monomer, i.e., Ce@pGOx_0, the photoluminescence intensity of Ce@pGOx_3 increases 3.2 times for the same cerium concentration, evidencing a significant boost in the shining intensity of the Ce(III) reporter upon coordination to phosphates (Figure 3A). Therefore, the significance of the polymeric component in this system is twofold: besides providing a convenient scaffold to retain, accommodate, and concentrate the Ce(III) cations, it fosters the optical response of the reporters.

Furthermore, we tested the influence of the composition of the hybrids and the environment on the fluorescence emitted by the chemobiosensor. Setting the Ce (III) seeding concentration to 0.2 mM, we observed that Ce@pGOx_3 sample emitted the highest fluorescence intensity (Figure 3B). Plus, we observed that the fluorescence of the system increased with the cerium concentration up to 0.6 mM (Figure S8). Unfortunately, as mentioned before, hybrids synthesized at >0.2 mM were discarded due to the formation of aggregates that might eventually disturb the glucose detection measurements.

We explored the possibility of decorating the polymeric mantel with distinct functional groups, *i.e.*, carboxylic acids and imidazole groups, that are broadly used for metal–ligand coordination.^32^ Imidazole and carboxyl groups were incorporated introducing vinyl imidazole and carboxyethyl acrylamide monomers in the polymerization reaction, giving rise to iGOx and cGOx nanogels, respectively (see the Supporting Information for further details). We observed that chemobiosensors assembled from pGOx hybrid shined with more efficiency than those synthesized from iGOx and cGOx nanogels, which showed 4.9 and 2.6 times less intensity than Ce@pGOx sample, respectively. Therefore, these results confirm the potential use of MAEPm as a fluorescence intensity enhancer ligand of cerium ions (Figure S9). Further, we demonstrated that Ce cation showed the best performance as a fluorescent reporter compared to other lanthanide metals such as Praseodymium and Terbium (Figures S10 and S11). Finally, we determined a Tris concentration of 5 mM and a pH of 8.0 as the optimum conditions that maximized the fluorescence of the chemobiosensor (Figure S12).

### Detection of Glucose: Mechanism and Catalytic Properties of the Hybrid Material

**Figure 4:**
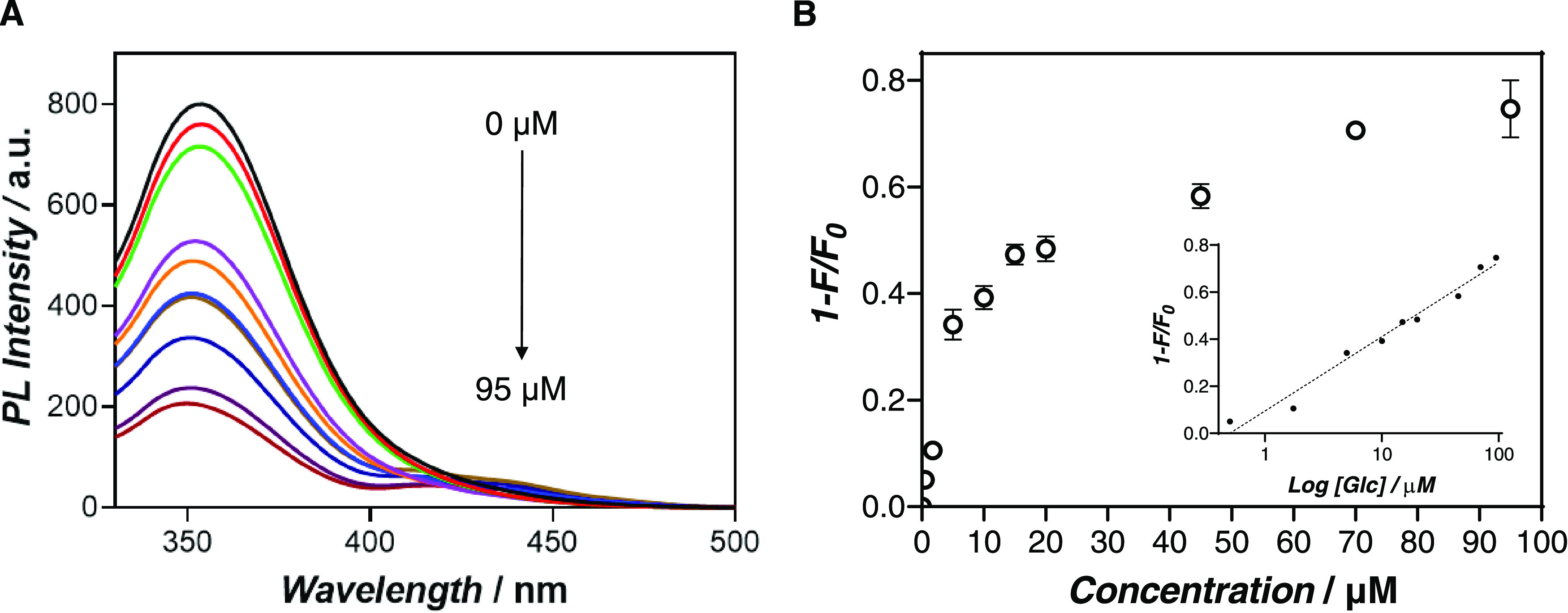
Sensitivity evaluation of Ce@pGOx nanogels as a glucose biosensor. (A) Photoluminescence intensity spectra of the chemobiosensor after incubation with concentrations of glucose from 0 to 95 μM. (B) Plot of the fluorescence quenching and the linear representation of the curve (inset) calculated from fluorescence values measured in (A).

We investigated the potential of Ce@pGOx nanogels as glucose biosensors. Considering the aggregation behavior of the system at a high concentration of Ce (III), sample Ce@pGOx_3 synthesized at a concentration of cerium nitrate of 0.2 mM was selected as optimal to achieve soluble and homogeneous chemobiosensors. Interestingly, we observed that the photoluminescence intensity of Ce@pGOx decreased upon the addition of glucose. This self-reporting hybrid material oxidizes the glucose and produces equimolar amounts of hydrogen peroxide, acting as a static fluorescence quencher (Figure S13). This reaction intermediate immediately switches off the fluorescence of the hybrid, which intensity drop can be correlated to the amount of glucose in the medium (Figures 4A and S14). An optimal incubation time of 30 min was assessed by the measurement of the temporal dynamics of the fluorescence response (Figure S15). In addition, the stability of Ce@pGOx in water was demonstrated to be very satisfactory, with no significant loss of fluorescence for at least 4 months (Figure S16).

The system exhibits a good linear correlation in the range of 0–95 μM with a correlation coefficient (*R*^2^) of 0.97 (Figure 4B). The limit of detection (LOD) was estimated at 73.37 nM according to the 3σ rule (eq 1). Importantly, compared to other in-solution protocols in which the enzyme and the Ce salts are separately co-added to the sample, the confined system allows similar sensitivities for the detection of the analyte with significantly less cerium content (8 vs 0.13 mM measured according to ICP-MS for free and confined systems, respectively).^8,9^ Moreover, compared to other glucose-measuring methodologies based on electrochemical, colorimetric, fluorometric, or Raman readouts (Table 2), our methodology showcases very high sensibility and high apparent recovery rates.

**Table 2:** Reported Materials for the Detection of Glucose in Tears^a^

method	material	linear range (μM)	detection limit (nM)	apparent recovery^b^ (%)	RSD^c^ (%)	refs
electrochemical	GDH	40–6200			15	(33)
electrochemical	GDH-modified		220 000			(34)
electrochemical	PTB-GOx	75–7500	22 200		2.58	(35)
colorimetric	GOx-HRP	100–1000	50 000	95	2.9–9.5	(36)
colorimetric	GOx-HRP	20–4000	14 000	94.3–98.0	<3.2	(37)
colorimetric	CNP-PEG-GOx	100–600		111.1–166.6	>10	(38)
fluorometric	PS@C6@PtP-GOx	100–2000	25 000		1.5–9.0	(39)
Raman (SERS)	GMXeP	1–50	390		11.7	(40)
electrophoresis	GOx-PLDz	10–100	5000		11.25	(41)
fluorometric	Ce@pGOx	0.5–95	73.37	99.7–100	2.2–20	this study

a The linear range, detection limit, apparent recovery, and the relative standard deviation (RSD) of each material are disclosed.

b Detected-added ratio in percentage.

c Relative standard deviation.

Therefore, we have demonstrated that the rational tailoring of the interface between two functional entities using tunable polymeric hydrogels can be applied to enhance their cross-interaction, thereby maximizing the performance of the chemoenzymatic systems.^42^ This configuration facilitates the fabrication of sensitive and self-reporting chemobiosensors. Of note, we have successfully applied our configuration to an alcohol oxidase enzyme (AOx, from *C. Boidinii*) for the detection of methanol in solution via fluorescence readout. (Figure S17).

**Figure 5:**
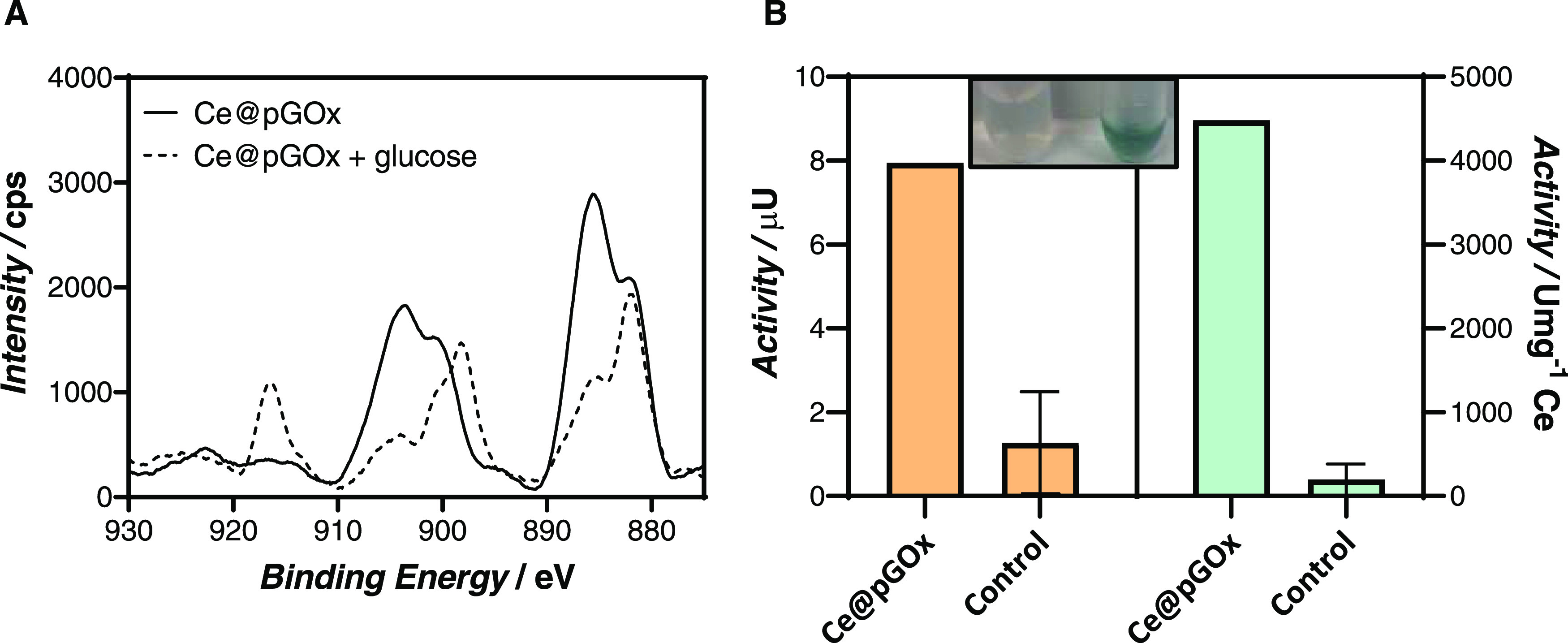
(A) Ce 3d XPS spectra of Ce@pGOx nanogels upon reaction with 100 mM glucose (black dashed line) and absence of glucose (black line). (B) Catalytic activity measurements of Ce@pGOx nanogels. The activity of the hybrid material is represented in μU (nmol glucose min^–1^) in orange and in terms of specific activity (Umg^–1^, μmol glucose min^–1^ mg^–1^) of Ce in blue. The inset displays the changes of color upon peroxidation reaction. The activity of Ce control was represented as a reference. The reaction was monitored by measuring the absorbance of oxidized ABTS at 416 nm.

We observed that the addition of glucose to the Ce@pGOx sample triggered the evolution of the color of the solution, which turns orange after several minutes. This observation might be due to the oxidation of Ce(III) to Ce(IV), which, in turn, might be responsible for the drop of fluorescence in our system. To ascertain our hypothesis, we unveiled the oxidation states of the cerium ion before and after the addition of glucose to the sample by X-ray photoelectron spectroscopy (XPS). As observed in Figure 5A, the Ce 3d main peak is characterized by two multiplets splitting (v and u) corresponding to the spin–orbit split 3d_5/2_ and 3d_3/2_.^43−45^ The four peaks resolved from this pair spin–orbit doublets (v′, u′, v°, and u°) are attributed to Ce^3+^. In contrast, the sample that contained glucose during the reaction displayed two additional peaks, a strong peak at 898 eV (v‴) and another peak at 917 eV, which corresponds to the fingerprint of Ce (IV) oxidation state. According to these results, it seems that the proximity of the active center of the enzyme to the cerium complex, situated on the surface of the nanogel, and the concomitant high local concentration of hydrogen peroxide around the polymeric counterpart, allows a rapid oxidation of the cerium compared to other nonintegrated systems.^46−48^

Interestingly, we observed that our system, besides its reporting capabilities, exhibits catalytic properties. The catalytic profile of Ce-based nanomaterials (nanoenzymes) has been broadly studied showing peroxidase, oxidase, catalase, superoxide dismutase (SOD), phosphatase, and haloperoxidase mimetic properties.^18,49,50^ Therefore, we monitored the oxidation ability of Ce@pGOx hybrids using ABTS as a colorimetric detection method (Materials and Methods section). Gratifyingly, we observed that, after several minutes of lag time (Figure S18), in which, presumably, Ce(III) is converted to Ce(IV) in the presence of glucose, the ABTS is clearly oxidized, giving rise to a blue-colored solution (Figure 5B). Remarkably, the oxidation of ABTS does not work in the absence of glucose, revealing that the Ce (IV) cation is responsible for the oxidase activity.^51,52^

### Use of Chemobiosensors for the Detection of Glucose in Tear Samples

To check the possibility of utilizing our system to detect the presence of glucose in fluid samples, i.e., tear fluids, we first determined its selectivity and robustness in the presence of distinct analytes. We proved that the photoluminescence emission was not affected in the presence of other disaccharides (maltose, sucrose, cellobiose, and lactose) (Figure S19A). In contrast, the fluorescence was detrimentally affected upon the addition of xylose, as it can be oxidized by the GOx enzyme, but not affected in the case of fructose and galactose.^53^ Additionally, the effect of other small molecules such as ascorbic acid and some amino acids (Arg, Gly, Ala, and Cys) was tested. Only the fluorescent ascorbic acid triggered a significant effect on the optical properties of the chemobiosensor, by reducing the fluorescence readout of the sample by 25% (Figure S19A). Also, we tested the robustness of the system in the presence of divalent metals (Mg^2+^ and Ca^2+^) and different salt concentrations usually present in biological samples (CH_3_COOK, NaCl, KCl, and NaH_2_PO_4_), with no significant changes in the fluorescence intensity (Figure S19B).

**Figure 6:**
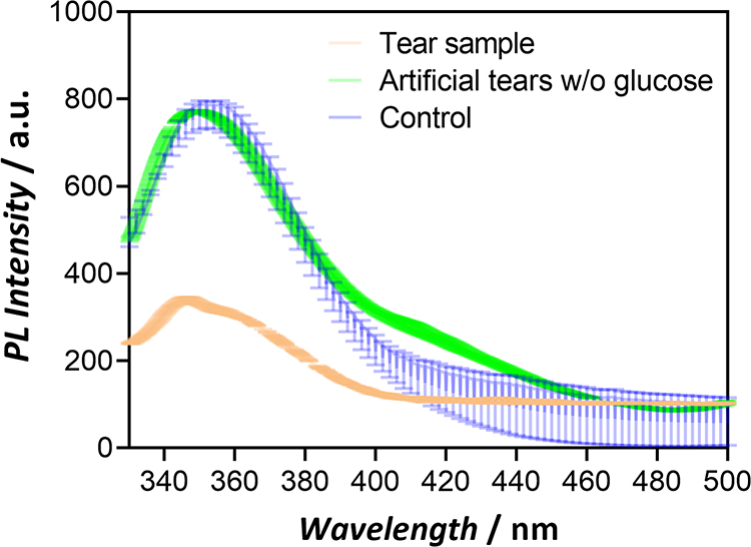
Photoluminescence spectra of Ce@pGOx in the presence of a solution with and without glucose at 40 μM.

Finally, its potential applicability for its use to quantify glucose in artificial tear samples was evaluated. As a first step, the accuracy of the system was assessed in artificial tear mixtures. Importantly, we observed that the composition of the tears has a slight effect on the intrinsic fluorescence intensity of the chemobiosensor, which underestimates the measured glucose concentration by ca. 4.5 μM in tears samples when applying the calibration curve displayed in Figure 4B (Figure S20 and Table S2). Therefore, considering the effect of the matrix, high apparent recoveries (99.7–100%) were achieved in the measured range (10–50 μM), which are comparable with other systems reported, but with significant improvement in the LOD (Table 2). When human tear fluid solution (4.5× dilution) was tested, the fluorescence intensity of the chemobiosensor was significantly reduced after 30 min of incubation (Figure 6). Our system measured a final glucose concentration of 30.9 ± 2.1 μM in the diluted sample, which represents a final glucose concentration of 0.031 g L^–1^ in the fluid.^54^ These results reflect the high potential of our chemobiosensor to detect and quantify the glucose level in human fluids.

## Conclusions

In conclusion, we have demonstrated that the confinement of distinct functional entities that work together in a concurrent manner is a good approach for the fabrication of effective chemosensing systems. The arrangement of the active (bio)materials in the nanospace allows the detection of analytes with a very high efficiency and less concentration of the reporter.^8^ The high specificity and the robustness of this chemobiosensor make it suitable to measure glucose concentrations, even in the presence of different amino acids, salts, and di- and monosaccharides. Interestingly, our hybrid turned out to be catalytic even at very low cerium concentrations present in the polymeric shell. This approach opens a straightforward route to the synthesis of a lanthanide-based biosensor with high sensitivity and catalytic properties. In summary, we report a useful methodology based on the utilization of polymeric architectures that allow the compartmentalization and stabilization of distinct functional (bio)materials for the sake of one-pot concurrent detection systems.

## Supporting Information Available

Further characterization of the chemobiosensor (pGOx) by SDS-PAGE, FPLC, thermal denaturation (circular dichroism), and SEM, and additional fluorescent and kinetic measurements (PDF)

## Abbreviations Used

PBS: phosphate-buffered saline
GOx: glucose oxidase
pGOx: GOx nanogels with phosphate moieties
Ce@pGOx: cerium-laden pGOx
DLS: dynamic light scattering
SEM: scanning electron microscopy
SEC: size exclusion chromatography
FPLC: fast protein liquid chromatography
ATR-FTIR: attenuated total reflection–Fourier transform infrared spectroscopy
SDS-PAGE: sodium dodecyl sulfate–polyacrylamide gel electrophoresis
CD: circular dichroism
LOD: limit of detection
ABTS: 2,2′-azino-bis(3-ethylbenzothiazoline-6-sulfonic acid)
SOD: superoxide dismutase
AOX: alcohol oxidase
Bpy: bypyridine
ATP: adenosine triphosphate
RSD: relative standard deviation
GDH: glucose dehydrogenase
PTB: poly(toluidine blue O)
CNP: cerium oxide nanoparticles
PEG: poly(ethylene glycol)
PS: polystyrene particles
C6: coumarin 6
PtP: platinum meso-tetra(pentafluorophenyl)porphyrin
GMXeP: gold nanoparticles with MXene nanosheets loaded on paper
PLDZ: pistol-like DNAzyme
