# Supplementary material for: Metal–Organic Enzyme Nanogels as Nanointegrated Self-Reporting Chemobiosensors
Source: ACS Appl Mater Interfaces. 2022 Jun 8;14(24):27589–98. doi: 10.1021/acsami.2c04385 (PMC9227723; doi:10.1021/acsami.2c04385)
Supplement: Supplementary file 1 — am2c04385_si_001.pdf [file am2c04385_si_001.pdf]

Supporting Information

Metal-Organic Enzyme Nanogels as Nanointegrated Self-reporting Chemobiosensors

Daniel Sánchez-deAlcázar[1], *Andoni Rodriguez-Abetxuko*[2], *Ana Beloqui*[1,3,*]

[1] POLYMAT and Department of Applied Chemistry, Faculty of Chemistry, University of the Basque Country UPV/EHU, E-20018 Donostia-San Sebastián, Spain

[2] CIC nanoGUNE, Basque Research and Technology Alliance (BRTA), Tolosa Hiribidea 76, E-20018 Donostia-San Sebastián, Spain

[3] IKERBASQUE, Basque Foundation for Science, Plaza Euskadi 5, E-48009 Bilbao, Spain

Corresponding Author

*E-mail: ana.beloquie@ehu.eus

1. Synthesis and characterization of pGOx nanogels

Single enzyme nanogels used for the fabrication of pGOx samples were characterized by Sodium Dodecyl Sulphate-Polyacrylamide Gel Electrophoresis (SDS-PAGE), Fast Protein Liquid Chromatography (FPLC), circular dichroism, and Scanning Electron Microscopy (SEM).

1.1. Size analysis by electrophoresis

The size of nanogels was measured by SDS-PAGE gels at 10% (Figure S1). The electrophoretic mobility of plain GOx correspond with ca. 70 KDa (lane 2) whereas the nanogels were retained in the upper part of the gel (lane 1). The increased size of the nanogel reduce the mobility through pores of acrylamide.

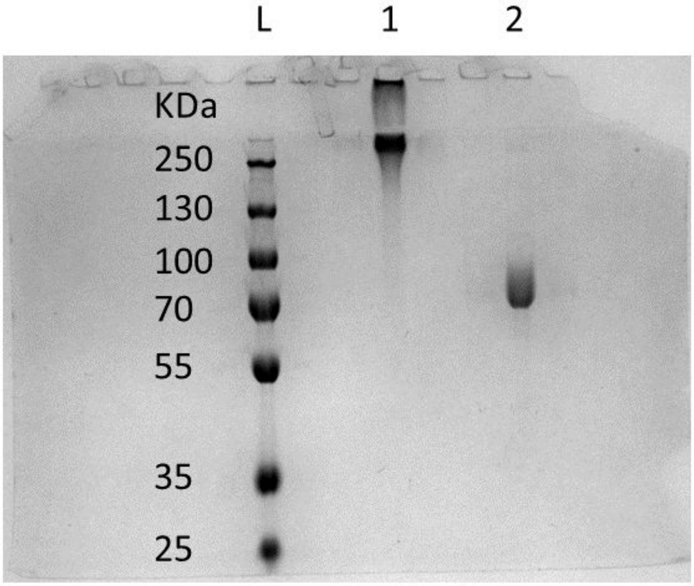

Figure S1. SDS-PAGE gel of ladder (L), pGOx nanogels (1), and plain GOx enzyme (2).

1.2. Size analysis by Size Exclusion Chromatography (SEC)

Fast Protein Liquid Chromatography (FPLC) was performed to determine the difference size of pGOx nanogel and plain GOx enzyme (Figure S2). GOx enzyme displayed a narrow peak which maximum is centred at 11.2 mL. As shown in the figure, pGOx nanogel showed a broad peak centred at 10 mL approximately, with two maxima due to the size polydispersity of nanogels.

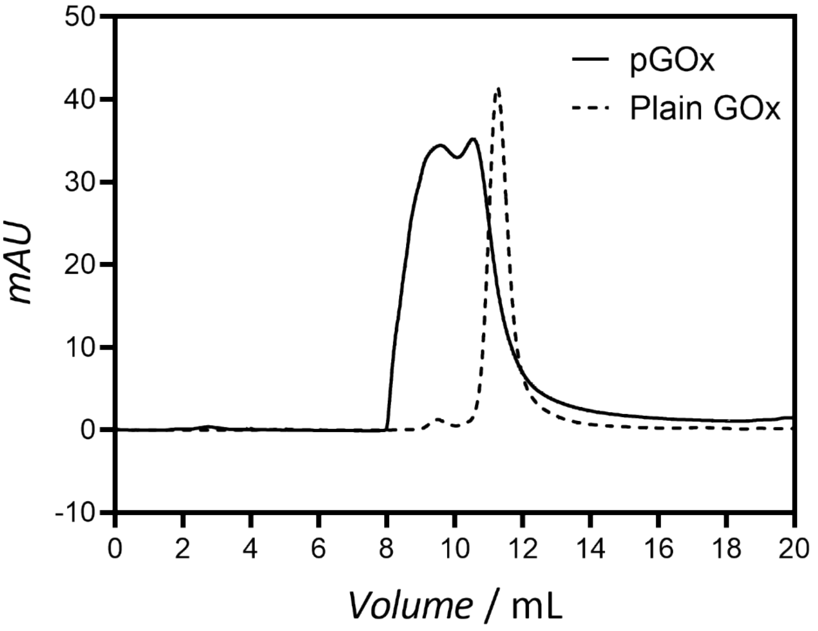

Figure S2. FPLC chromatogram of pGOx nanogels and plain GOx enzyme.

1.3. Thermal stability analysed by circular dichroism

Thermal denaturation was performed with circular dichroism technique (Figure S3). Dichroic signal is monitored at 222 nm (corresponding to the dichroic signal of α-helix) as function of the temperature (in the range 20 – 90 □C). The intensity of the dichroic signal of plain protein drastically decreased, showing a melting temperature (T_m) of less than 60□C, which is the normal values of T_m for the most of the proteins[1–4], losing completely the secondary structure of the protein at 90□C. In contrast, the protective polymeric mantle of encapsulated protein avoids denaturation of the protein (sheltering effect, maintaining the stability in the range of temperature analysed. It is worth mentioning that there is a small change in the dichroic signal at high temperatures (above 50□C), but it is quickly stabilized in all the range from 60 to 90□C. This slight change

in the dichroic signal was attributed to tiny changes in the conformation of the protein inside of the polymeric capsule.

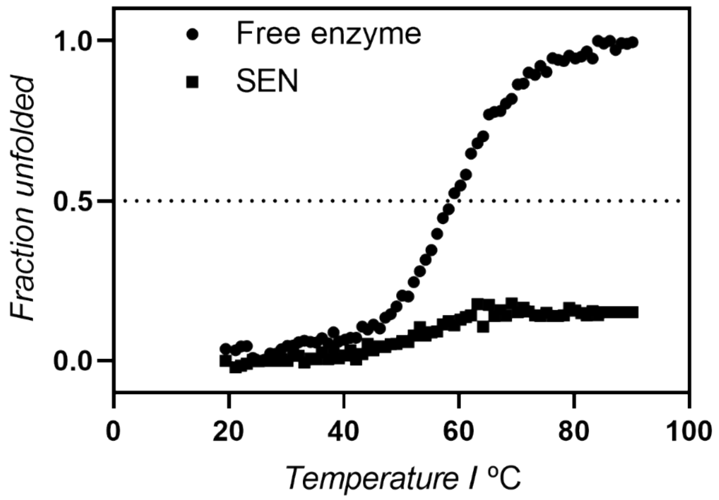

Figure S3. Monitoring of the thermal denaturation of free enzyme and single enzyme nanogels by circular dichroism.

1.4. Kinetic characterization

Activity characterization of plain GOx and encapsulated (pGOx) was performed. GOx/HRP/ABTS assay. The experiment was carried out measuring the change of absorbance at 416 nm over the time, monitoring the oxidation of ABTS. Glucose at 80 mM (approximately 4 times the K_M) was employed as a substrate for the production of hydrogen peroxide.

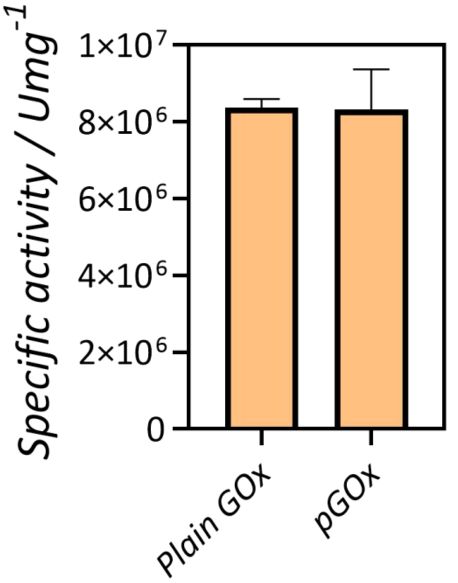

Figure S4. Specific activity of plain GOx and pGOx.

2. Analysis by Inductively coupled plasma mass spectrometry (ICP-MS)

Similar ratios of Ce/pGOx_3 nanogels were determined by ICP-MS for prepared samples (Table S1). Samples prepared with a seeding concentration of 0.2 mM Ce(III) and 1.25 µM nanogels showed 76% Ce loading.

Table S1. ICP-MS measurements of Ce@pGOx nanogels.

Sample	[Ce]/ µgL^{-1}	[NGs]/ µgmL^{-1}	Ce/ µg	NGs/ µg	Ce/µmol	Protein/ µmol	Ratio Ce/NG
1	159.90±7.44	0.77	0.1599± 0.0074	0.77	0.00114± 0.00005	4.8 10^{-6}	237.5±10.4
2	1773.92±75.70	7.7	1.7739± 0.0757	7.7	0.01266± 0.00054	4.8 10^{-5}	263.7±11.2
3	7689.22±333.46	38.5	7.6892± 0.33345	38.5	0.05488± 0.00238	2.4 10^{-4}	228.7±9.9
						**Mean**	**243.3±18.2**

3. Microscopy analysis

Ce@pGOx nanogels using high magnification (Figure S5). pGOx nanogels mixed with hight amount of Ce(III) lead to the self-assembly of nanoparticles (>0.8 mM of Ce(III)) (Figure S6).

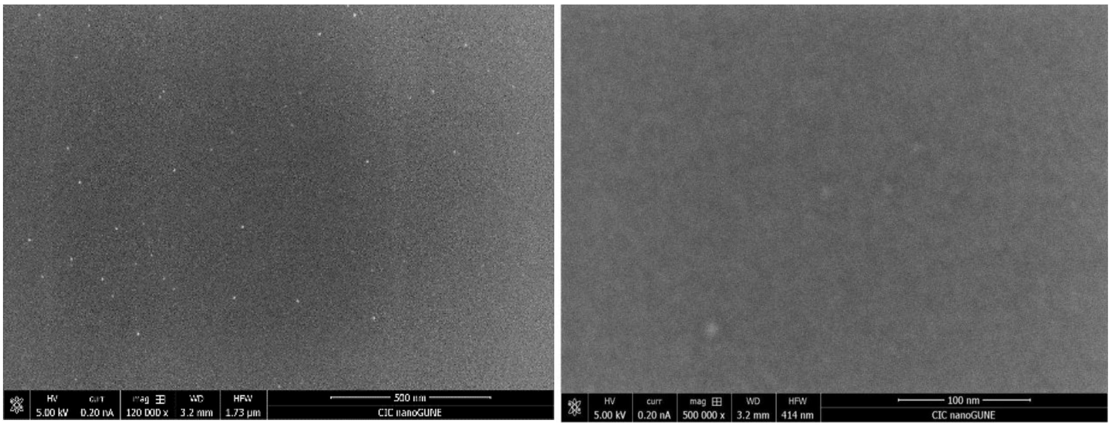

Figure S5. SEM images of Ce@pGOx nanogels at cerium concentration of 0.2 mM. High magnification (120,000x & 500,000x).

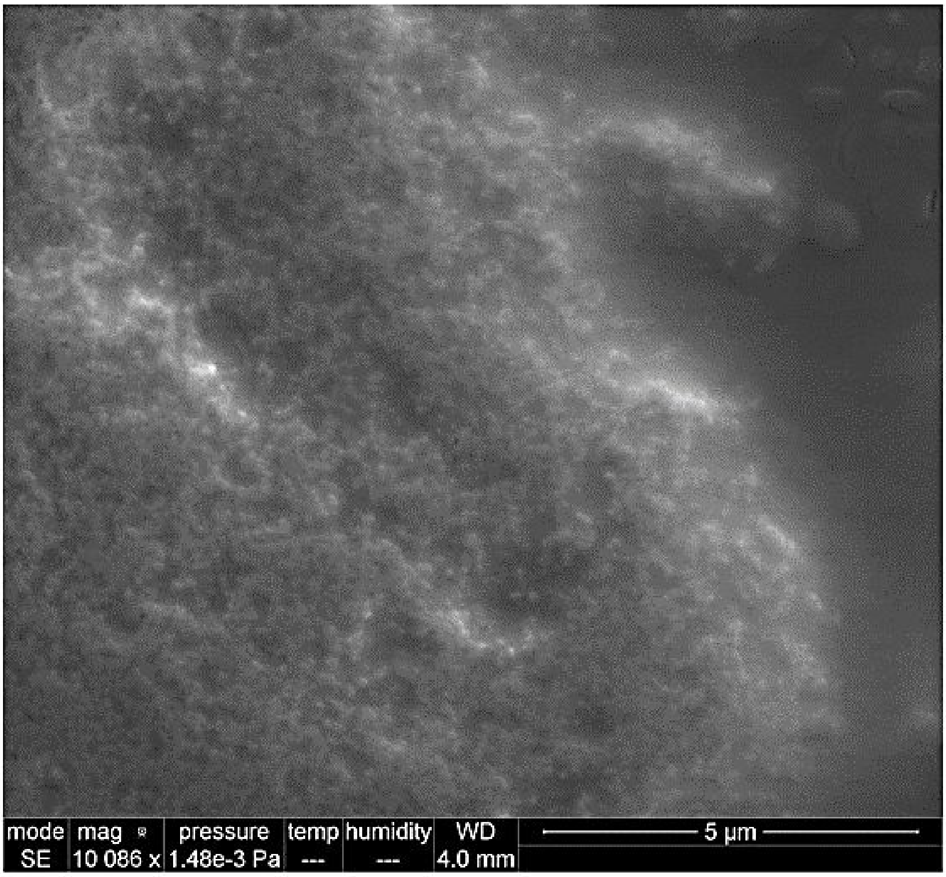

Figure S6. SEM image of Ce@pGOx nanogels at cerium concentration of 0.8 mM

4. Photoluminescence properties of Ln@pGOx

4.1. Reproducibility of the synthesis

Reproducibility was evaluated by measuring fluorescence intensity of the chemobiosensor using different batches, resulting in an average value of 504±11.1 and a relative standard deviation (RSD) of 2.2%.

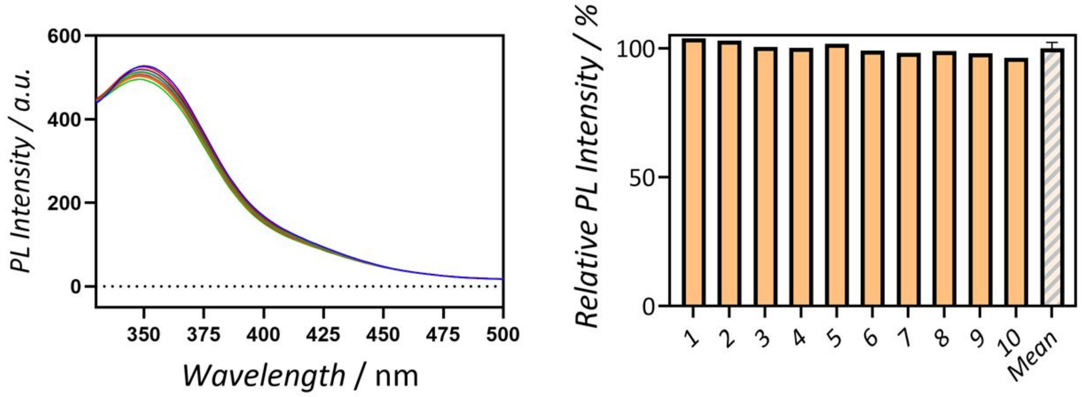

Figure S7. Reproducibility studies of Ce@pGOx nanogels.

<u>4.2. Photoluminescence of Ce@pGOx: Ce and MAEP:protein ratios optimization</u>

Non-purified samples of Ce@pGOx nanogels was measured by fluorescence (Figure S7). In a first approach, different MAEPm:protein molar ratios were explored with different concentration of Ce(III), i.e., from 0 to 0.8 mM. Similar intensity emissions were obtained for the same ratio, but increased intensities were found as cerium concentration boosted. Concentrations of 0.6 and 0.8 mM resulted in similar signals, thus reaching saturation signal. Concentration above 0.8 give rise to aggregation of nanogels forming metal-organic enzyme aggregates.

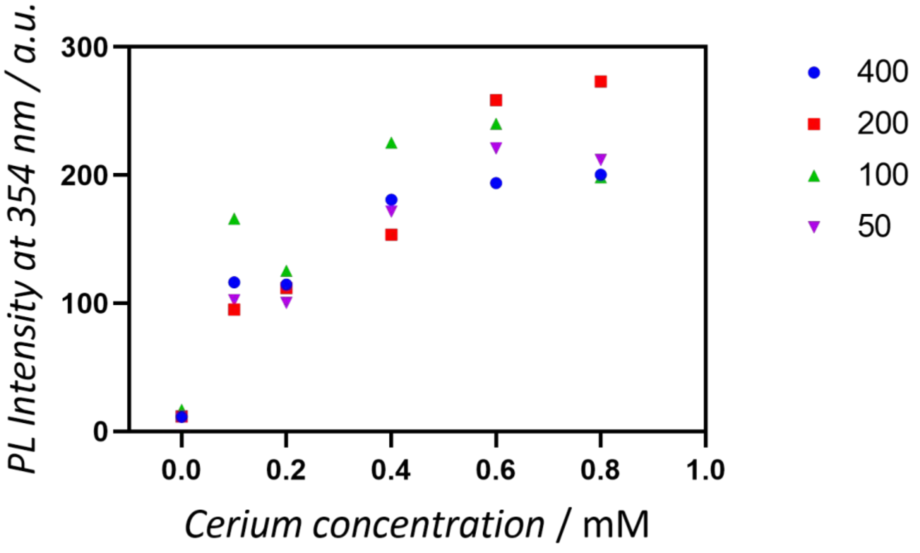

Figure S8. Effects of cerium concentration in the photoluminescence properties of Ce@pGOx nanogels represented against the MAEPm:GOx molar ratio, i.e., 400, 200, 100, and 50 (non-purified samples from excess of cerium).

4.3. Photoluminescence of Ce@pGOx: Effect of co-polymer composition

Different co-monomers, i.e., carboxyethyl acrylate (CEAm) and vinyl imidazole (VIm), with potential to coordinate Ce(III) were used for the fabrication of distinct GOx nanogels, i.e., cGOx and iGOx, respectively. Samples were evaluated by emitted fluorescence (Figure S8), showing less binding capacity against Ce(III) than MAEPm.

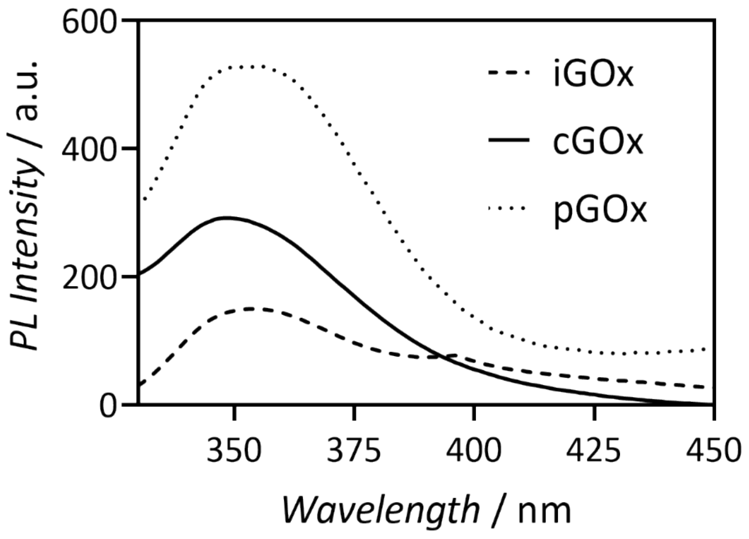

Figure S9. Influence of functional groups within the polymeric shell, vinyl imidazole (VIm), carboxyethyl acrylate (CEA), and monoacryloxyethylphosphate (MAEPm), on the fluorescence intensity of the built-in chemobiosensor.

4.4. Photoluminescence of Ln@pGOx: effect of Praseodymium (Pr) and Terbium (Tb)

The fluorescence of chemobiosensors fabricated with Pr and Tb was evaluated (λ_{ext}: 310 nm).

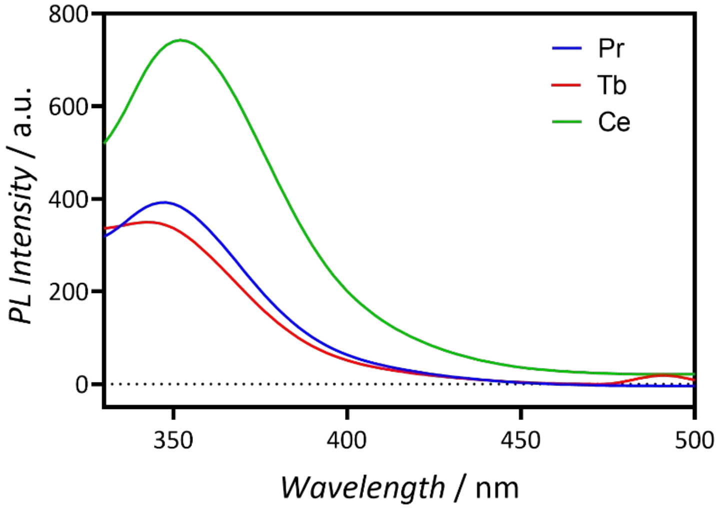

Figure S10. Fluorescence measurements for different lanthanide metals.

We assessed the performance of the Tb@pGOx hybrids as glucose biosensors. However, we discarded the chemobiosensors as they do not show a linear response in presence of glucose.

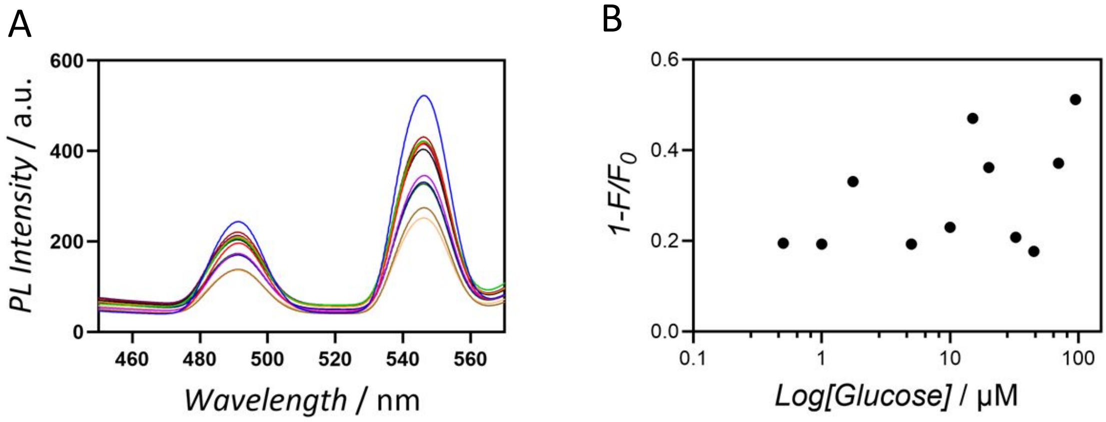

Figure S11. Sensitivity evaluation of Tb@pGOx nanogels as glucose biosensor. **A**. Photoluminescence intensity spectra of the chemobiosensor after incubation with concentrations of glucose from 0 to 95 µM. **B**. Plot of the fluorescence quenching.

4.5. Effect on the photoluminescence of Ce@pGOx: pH

Concentration of protons is a parameter that might have an influence in the optical properties of the system. Therefore, the fluorescence intensity was evaluated at 354 nm at different pH values, in order to optimize the optical response of the sensor. Tris-HCl buffer 5 mM at pH 7, 8 and 9, and 5 mM MES buffer at pH 5 and 6 were tested under same reporter concentration.

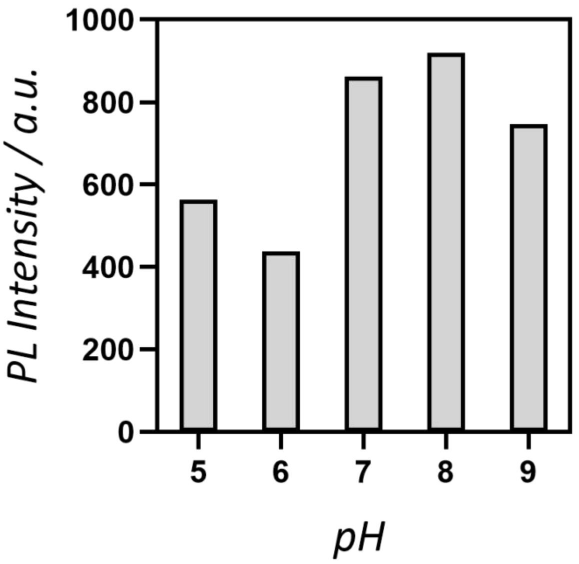

Figure S12. Influence of the pH in the fluorescence emission of the chemobiosensors.

5. Quenching mechanism

Quenching mechanism was evaluated by studying the behaviour with temperature. Static quenchers form a weak complex with the cerium in which just applying high temperature this induce the dissociation, being the quenching less effective. In this regard, we demonstrated that fluorescence decay is lower at 37 degrees than 25 degrees (Figure S13), showing that the behaviour is static, forming the hydrogen peroxide a weak complex with

the Ce(III). Moreover, this mechanism would explain the lag-time in the activity results as well as the mechanism purposes for the catalysis (ref. https://doi.org/10.1038/am.2013.88).

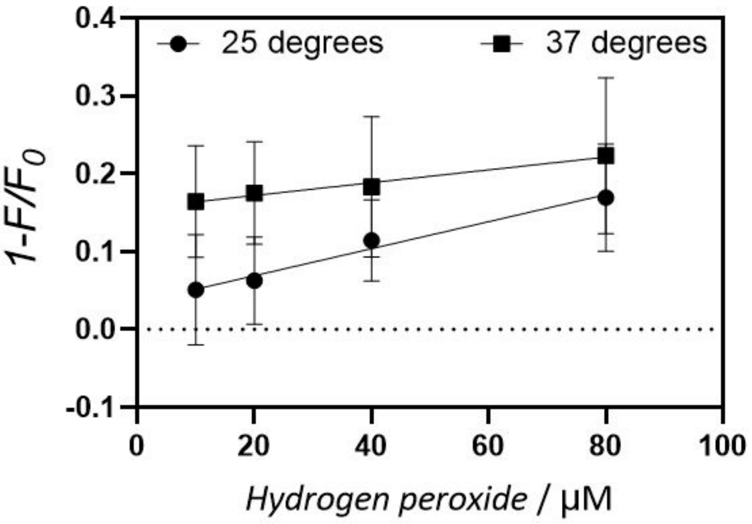

Figure S13. Fluorescence quenching of hydrogen peroxide at different concentration.

6. Sensing

The fluorescence of the chemobiosensors at different concentrations of glucose was studied. As observed, the produced hydrogen peroxide quenches the fluorescence of the system.

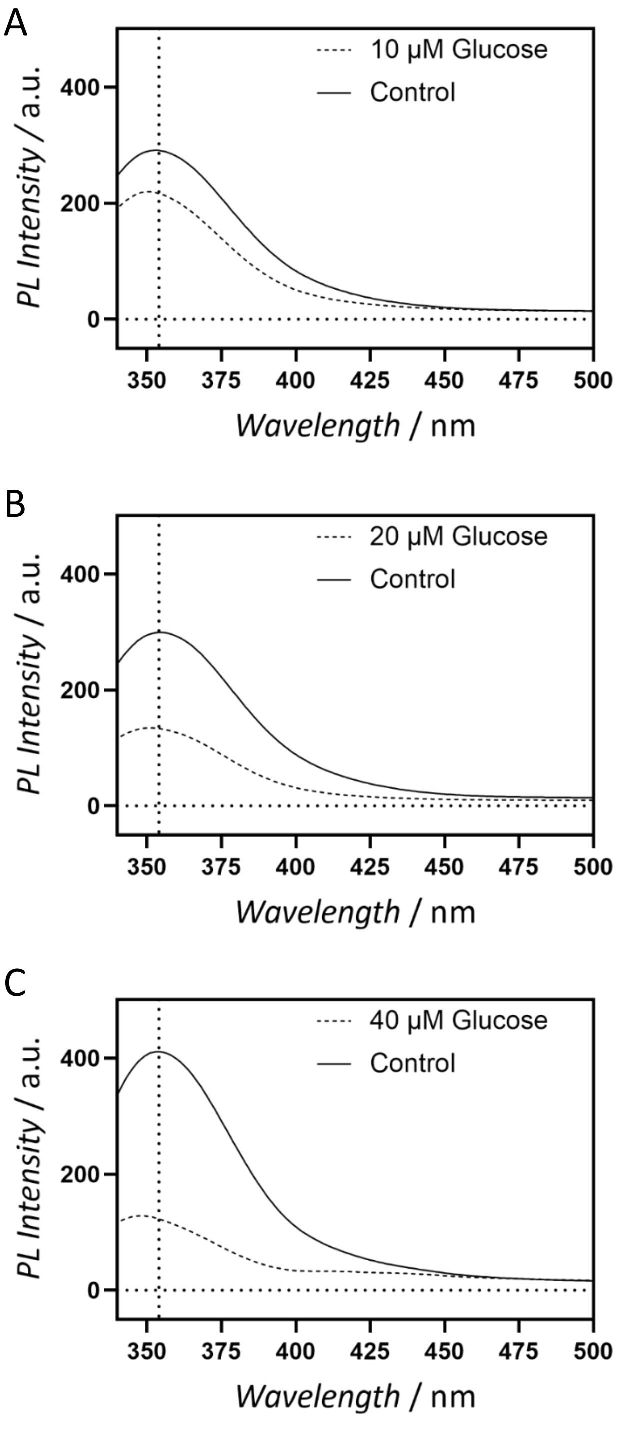

Figure S14. Effect of glucose concentration on the photoluminescence of the chemobiosensor. Three glucose concentrations were evaluated: A. 10 μM; B. 20 μM; C. 40 μM.

7. Time-course measurements

The decay of photoluminescence was measured in order to demonstrate the most suitable incubation time with substrate.

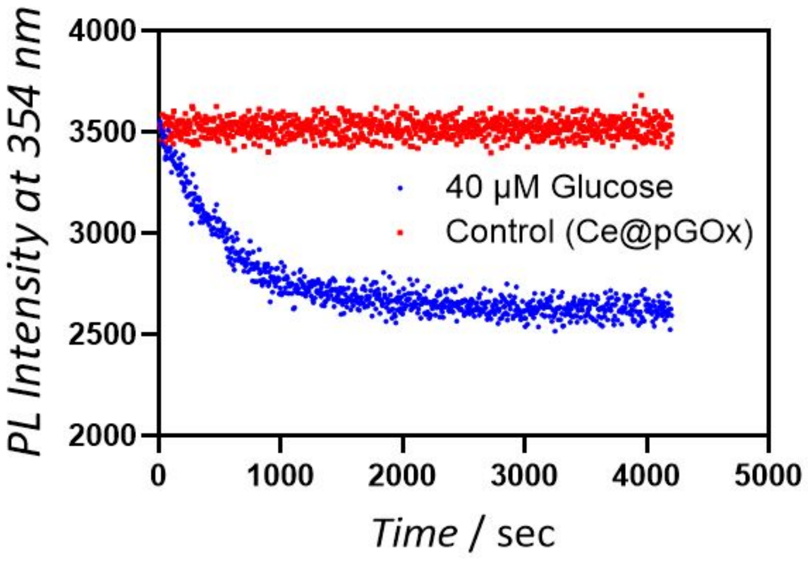

Figure S15. Time-course measurements of Ce@pGOx nanogels in presence and absence of glucose (40 µM).

8. Stability of the chemobiosensor

The stability of the chemobiosensor was evaluated measuring the fluorescence of a sample prepared four months ago compare with a sample recently prepared. The intensity of the elder sample was unaltered indeed.

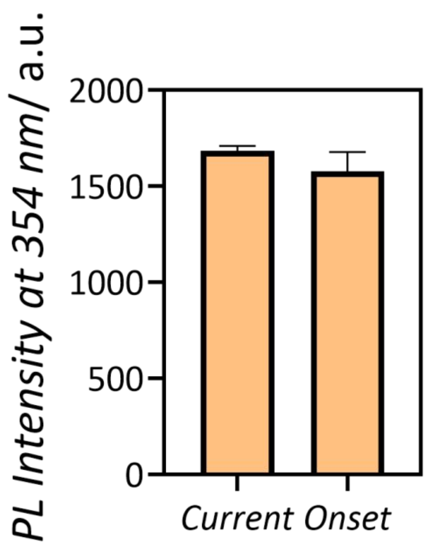

Figure S16. Photoluminescence intensity of Ce@pGOx measured four months ago and very recently.

9. Expansion of our configuration to the fabrication of other chemobiosensors

While the manuscript is focused on the development of a sensor of glucose, our configuration can be applied to the fabrication of other chemobiosensors, based on the encapsulation of assorted oxidases that release hydrogen peroxide as subproduct of the reaction. As example, we have applied the protocol described in the manuscript to fabricate alcohol-responsive chemobiosensors, throughout the synthesis of alcohol oxidase nanogels (AOx from *C. boidinii*), which interfase is decorated with Ce(III) ions. As depicted in the Figure S17, the optical properties of Ce@pAOx chemobiosensor are modified in presence of methanol in solution, which suggests that it is feasible to fabricate alcohol-biosensors using our configuration.

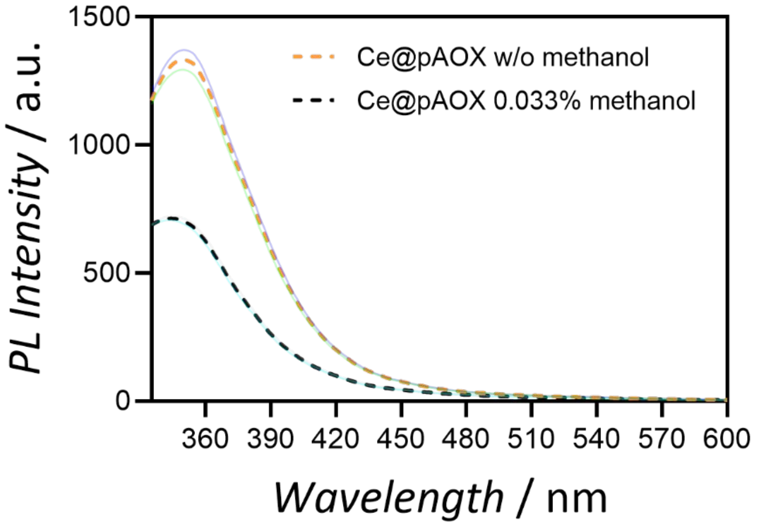

Figure S17. Photoluminescence spectra of Ce@pAOX nanogels in presence and absence of methanol at 0.033%.

10. Peroxidase-like activity of Ce@pGOx nanogels

Peroxidase activity of Ce@pGOx nanogels was measured. The peroxidase activity was measured using 2,2´-azino-bis(3-ethylbenzothiazoline-6-sulphonic acid) (ABTS) in sodium phosphate buffer (50 mM, pH 6) and 50 mM of glucose at 37 □C.

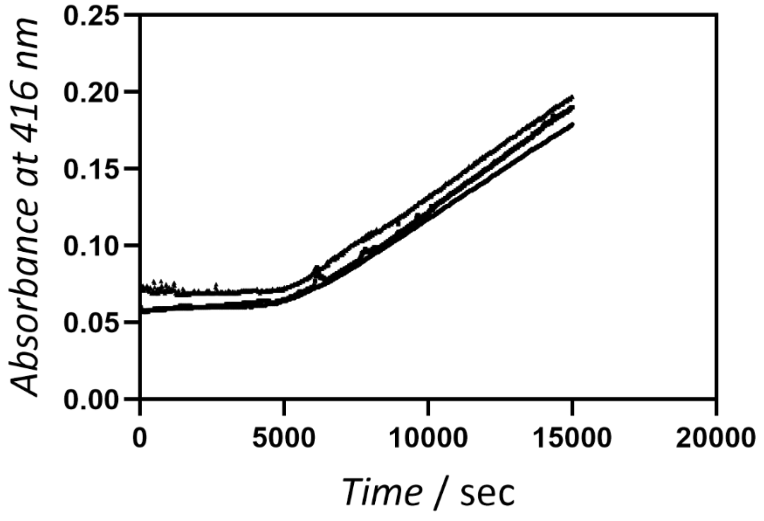

Figure S18. ABTS oxidation activity measurements of Ce@pGOx nanogels.

11 Fluorescence stability and selectivity of chemobiosensor

Fluorescence measurements of Ce@pGOx nanogels containing salts and sugars were evaluated.

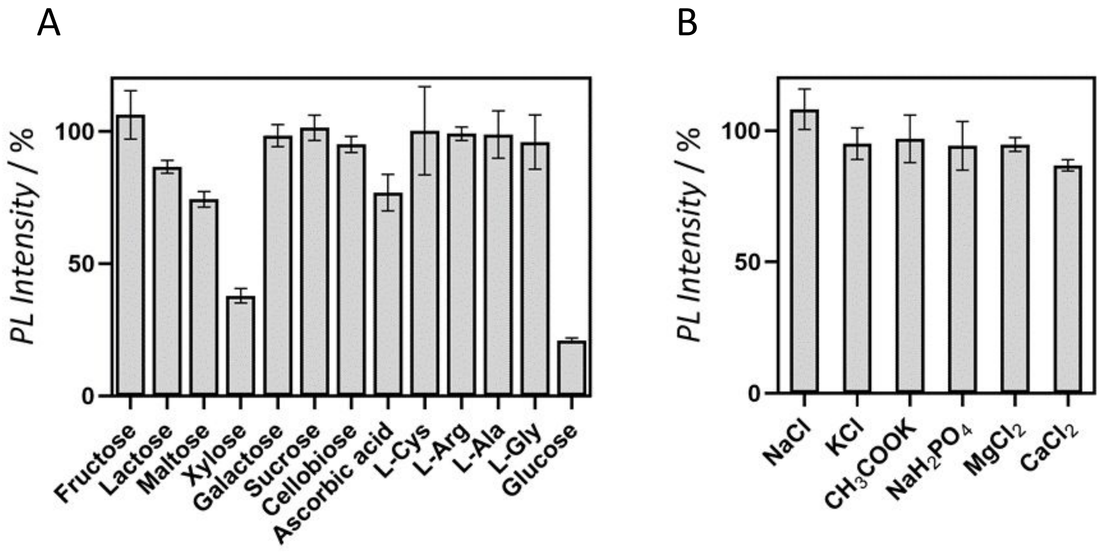

Figure S19. Relative photoluminescence emission of Ce@pGOx nanogels in presence of 30 mM of saccharides (A), and 100 µM of divalent metals and salts (B).

12. Accuracy and precision

Different amounts of glucose were spiked to the artificial tear solution to yield samples with 10, 40, and 50 µM of glucose (within the range of real tears). The concentration of glucose of these samples was measured with our fluorometric biosensor based on the calibration curve in Figure 4B. The results and parameters obtained are compiled in the **Table S2.** The results show that the glucose concentration measured in artificial tears was close to the reference value, but shifted by -4.5 µM. The representation of "measured glucose" vs "added glucose" showed a regression line of $y = 0.9973 (\pm 0.0001) x - 4.47 (\pm 0.02)$, with a slope very close to the ideal value of 1, which would reflect a fully accurate system. These results suggest that the error of around -4.5 µM may have arisen from some small differences in the fluorescence between the calibration curve (calculated in Tris buffer) and the measurement in tears mixture. In fact, Figure 6 of the manuscript shows a difference in the intrinsic fluorescence intensity of the chemobiosensor in Tris buffer (control) and in tears mixture in the absence of glucose. Considering the effect of the matrix on the chemobiosensor, we recalculated the recovery values, which fitted in the range of 99.7 – 100%. Moreover, the low relative standard deviation (RSD) of each of the measurements shows the high precision of Ce@pGOx.

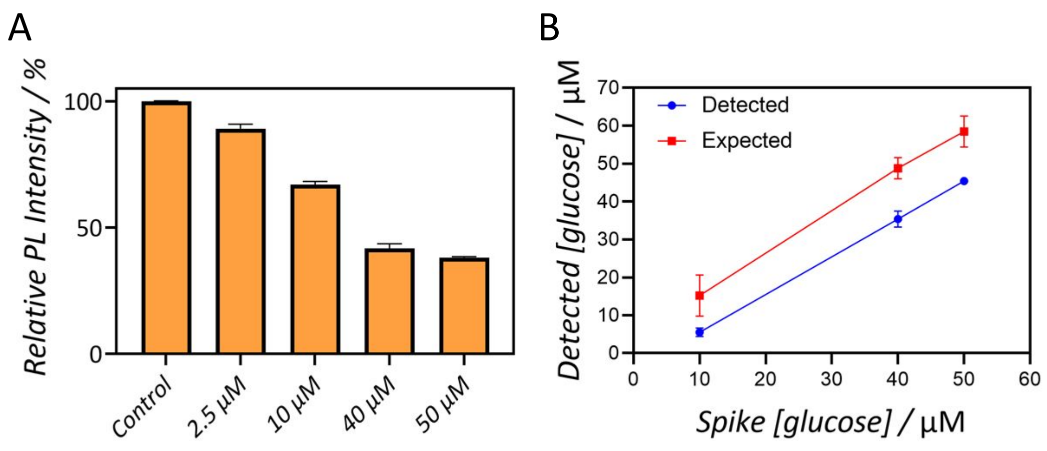

Figure S20. Evaluation of chemobiosensor with artificial tear samples. A. Relative photoluminescence intensity at 354 nm of Ce@pGOx in response to tears sample (10, 40, and 50 µM) after 30 min of incubation . B. Comparation of accuracy and precision of Ce@pGOx against commercial colorimetric methods for detecting glucose based on GOx/HRP assay.

Table S2. Accuracy and precision comparation of chemobiosensor with colorimetric method.

Sample	Added / µM	Detected / µM	Apparent recovery[1] / %	Corrected apparent recovery[2] / %	RSD[3] / %
2	10	5.5±1.1	55.0	100	20.0
3	40	35.4±2.1	88.5	99.7	5.9
4	50	45.4±1.0	90.8	99.8	2.2

[1]Detected-added glucose ratio in percentage. [2]Detected corrected by 4.5 factor-added glucose ratio in percentage. [3]Relative standard deviation.

REFERENCES

(1) Zoldák, G.; Zubrik, A.; Musatov, A.; Stupák, M.; Sedlák, E. Irreversible Thermal Denaturation of Glucose Oxidase from Aspergillus Niger Is the Transition to the Denatured State with Residual Structure. *J. Biol. Chem.* **2004**, *279* (46), 47601–47609. https://doi.org/10.1074/jbc.M406883200.

(2) Liu; Piccirilli; Liu; Li; Wang; Shen. Deciphering the Role of V88L Substitution in NDM-24 Metallo-β-Lactamase. *Catalysts* **2019**, *9* (9), 744. https://doi.org/10.3390/catal9090744.

(3) Chen, L.; Shukla, N.; Cho, I.; Cohn, E.; Taylor, E. A.; Othon, C. M. Sucralose Destabilization of Protein Structure. *J. Phys. Chem. Lett.* **2015**, *6* (8), 1441–1446. https://doi.org/10.1021/acs.jpclett.5b00442.

(4) Sanchez-deAlcazar, D.; Mejias, S. H.; Erazo, K.; Sot, B.; Cortajarena, A. L. Self-Assembly of Repeat Proteins: Concepts and Design of New Interfaces. *J. Struct. Biol.* **2018**, *201* (2), 118–129. https://doi.org/10.1016/j.jsb.2017.09.002.

(5) La Belle, J. T.; Adams, A.; Lin, C. E.; Engelschall, E.; Pratt, B.; Cook, C. B. Self-Monitoring of Tear Glucose: The Development of a Tear Based Glucose Sensor as an Alternative to Self-Monitoring of Blood Glucose. *Chem. Commun. (Camb).* **2016**, *52* (59), 9197–9204. https://doi.org/10.1039/C6CC03609K.
